# “I had given up on being a mother”: a survey of 183 women’s experience of transabdominal cerclage (TAC)

**DOI:** 10.1186/s12884-023-06001-w

**Published:** 2023-10-24

**Authors:** Jenny Carter, Joanne Deery, Manju Chandiramani, Andrew Shennan

**Affiliations:** 1 Department of Women and Children’s Health, School of Life Course and Population Sciences, King’s College London, London, UK; 2 UK TAC Support, Margate, UK; 3 Guy’s & St Thomas’ NHS Foundation Trust, London, UK

**Keywords:** Cerclage, Transabdominal cerclage, TAC, Preterm birth, Women’s experience, Survey

## Abstract

**Background:**

Transabdominal cerclage (TAC) is a relatively uncommon intervention for preventing preterm birth. This study aimed to investigate the experience of women who had undergone this procedure.

**Methods:**

The survey was designed in collaboration with a preterm birth studies public and patient involvement (PPI) group and ethical approval was granted by KCL BDM Research Ethics Panel (LRS-19/20-13205). Members of closed Facebook group, UK TAC Support, were invited to complete an online questionnaire about their experience of TAC, and pregnancies before and after having it placed. The survey was open between December 2019 and May 2020. Open and closed questions provided both qualitative and quantitative data for analysis, which was carried out using NVivo Pro 2020 v.1.4.1 qualitative data management software and SPSS Statistics 27 (IBM).

**Results:**

One hundred eighty-three participants completed the survey, having had TAC procedures carried out in 36 hospitals. Altogether, participants had experienced 287 preterm births (PTB) and late miscarriages (LM), equating to an average of 1.6 each (range 0–5), including 18 stillbirths. TAC was indicated in 123 (67%) for previous PTB and/or LM, 29 (16%) for cervical surgery and 31 (17%) for both. 151 (83%) TAC procedures were open, 32 (17%) laparoscopic. 86% (*n* = 157) were placed outside pregnancy. Of those placed in pregnancy, gestation at TAC ranged from 7 to 16 weeks. When comparing earliest pre- and post-TAC pregnancy gestation (excluding first trimester losses), median gestational weeks gained following TAC was 15.5 weeks (SD 6.89). Qualitative themes included: the struggle to get treatment; lack of TAC knowledge amongst clinicians; gratitude, hope and feeling protected; possible detrimental effects of TAC.

**Conclusions:**

This very high-risk group found having a TAC gave great reassurance and hope, and were very grateful to have found the care they needed. However, they often struggled to get this support, frequently due to lack of clinician awareness. This may improve following roll-out of NHS England’s Saving Babies Live Care Bundle and NHS commissioning guidelines for care of women at risk of PTB.

**Supplementary Information:**

The online version contains supplementary material available at 10.1186/s12884-023-06001-w.

## Background

Preterm birth is a major public health issue as it remains a major cause of neonatal mortality and long-term morbidity [1]. Efforts to address this include interventions to prevent preterm birth, or to at least extend gestation, include progesterone supplementation and cervical cerclage [2]. In cervical cerclage, a suture is placed around the cervix and tied. It is usually performed vaginally, but for some women, e.g., where there is little accessible cervical tissue following radical trachelectomy, transabdominal cerclage (TAC) may be offered. In this procedure, the cerclage is placed at the level of the internal cervical os, either through open or laparoscopic surgery. It has been shown to be highly effective in women at very high risk of preterm birth, when vaginally placed cerclage has previously failed [3]. As it is impossible to remove TAC vaginally, caesarean section is necessary to deliver the baby, however, it can remain in situ, and thus protect future pregnancies.

The incidence of transvaginal cerclage procedures is currently unknown, although it appears to be increasing. In our tertiary hospital, with around 6,000 maternities annually, the number of TACs carried out has increased from around one per month, 5 years ago, to approximately one per week. Overall, it remains relatively uncommon, and little is known about the experience from the perspective of women who have received it. We had heard, anecdotally from women referred to our preterm clinic, that many had had difficulties in procuring specialist preterm care after sometimes multiple pregnancy losses, and we wanted to explore this formally. This would allow us to increase awareness amongst clinicians and make recommendations for improving care in the future. In this paper, we present the findings from an online survey of members of UK TAC Support, a Facebook support group for women who believe their experience of previous pregnancy loss, preterm birth or radical cervical surgery, renders them suitable for this intervention. The group was set up in 2013 by four women with similar experiences, following their own struggle to find information and support. The aim of this study was to explore the experiences of members of this group. Our objectives were to explore: 1. Reasons for having TAC; 2. variation in TAC procedures (e.g. methods, anaesthetic use and concurrent interventions); 3. need for repeated TAC procedures; 4. women’s experience of seeking and receiving a TAC and 5. pregnancies before and after TAC.

## Methods

A questionnaire was devised in collaboration with the King’s Health Partners preterm birth studies public and patient involvement (PPI) group (see Additional file 1) and published using the online survey platform, JISC surveys. Ethical approval was granted by King’s College London’s BDM Research Ethics Panel (LRS-19/20-13205). Members of closed Facebook group, UK TAC Support, who had personal experience of TAC (*n* = 590) were invited to complete the online questionnaire about their experience of TAC and their pregnancies before and after having it placed. The survey was open between December 2019 and May 2020. An invitation to participate was posted by the group’s facilitator with a reminder posted on two further occasions. Quantitative data was analysed using SPSS Statistics 17 (IBM). Qualitative Description [4] informed iterative thematic analysis of the qualitative data collected in the free text question which was carried out using NVivo Pro 2020 1.4.1 qualitative data management software.

In order to explore differences in outcomes before and after TAC placement we undertook a sub-group analysis, excluding participants with no pre-TAC (*n* = 21) or post-TAC (*n* = 86) pregnancies and pregnancies ending before 14 weeks’ gestation. We also excluded 9 cases with erroneous data (i.e. dates likely to be the participant’s date of birth or obvious errors, e.g. “1930”, “2100”), and a further two where the cause of miscarriage was stated as intrauterine growth restriction (IUGR) or “Trisomy” (i.e. fetal chromosomal anomaly). If participants had had more than one pregnancy before and/or after the TAC, we compared the gestations of the earliest pregnancies in the appropriate time frame/s. Pre- and post-TAC pregnancy gestations were compared using paired samples t-test.

## Results

### Participant characteristics

Of the 590 eligible members, 183 (31.0%) respondents completed the survey, having had TAC procedures carried out in 28 NHS/public and 8 private hospitals throughout the UK and the Republic of Ireland. Altogether, participants had experienced 131 preterm births (PTB) and 156 late miscarriages (LM), equating to an average of 1.6 PTB/LM each (287/183, range 0–5). Of these, 18 babies were stillborn. Demographic characteristics are shown in Table 1.

**Table 1:** Demographic characteristics of all respondents, and those providing free text for qualitative analysis

	All respondents		Respondents providing free text for qualitative analysis	
	*n* =	*%*	*n* =	*%*
**Ethnicity**				
*Asian*	17	*9.3*	11	*9.5%*
*Black*	16	*8.7*	10	*8.6%*
*Mixed*	3	*1.6*	1	*0.9%*
*Other*	1	*0.5*	1	*0.9%*
*White*	146	*79.8*	93	*80.2%*
All	183		116	
**Age Group**				
*25 to 29*	26	*14.2*	13	*11.2%*
*30 to 34*	38	*20.8*	22	*19.0%*
*35 to 39*	74	*40.4*	50	*43.1%*
*40 to 44*	39	*21.3*	29	*25.0%*
*45 and over*	6	*3.3*	2	*1.7%*
All	183		116	

### Reasons for having TAC

The majority of women reported the reason for their TAC as previous preterm birth (PTB) or late miscarriage (LM) alone (67%, *n* = 123), while the indication for 29 (15.8%) was cervical surgery alone. 31 women (16.9%) reported both PTB/LM and cervical surgery. These findings are shown in Table 2.

**Table 2:** Indications for TAC

Indication for TAC	*n* =	*%*
Cervical Surgery	29	*15.8*
Previous late miscarriage or preterm birth	123	*67.2*
Both	31	*16.9*
All	183	

Of the 60 women who reported invasive cervical surgery as being at least one indication for their TAC, 16 (26.7%) had had a trachelectomy, the most radical procedure undertaken to remove cancerous cervical tissue, 29 (48.3%) had large loop excision of the transformation zone (LLETZ), 11 (18.3%) cone biopsy and 4 (6.7%) were unsure of the type of cervical surgery (see Table 3).

**Table 3:** Types of cervical surgery reported as indications for TAC

Type of cervical surgery	*n* =	*%*
Cone Biopsy	11	*18.3*
LLETZ	29	*48.3*
Trachelectomy	16	*26.7*
Unsure/unknown	4	*6.7*
Women reporting cervical surgery as indication for TAC	60	

### Variation in TAC procedures

Most TAC procedures were open (82.5%, *n* = 151), while 32 (17.5%) were carried out laparoscopically and the majority (85.8%, *n* = 157) were placed outside pregnancy. Of those placed in pregnancy, all were open procedures, and gestation at TAC ranged from 7 to 16 weeks. The majority of women reported that their TAC was placed by a consultant (90.7%, *n* = 166), five said it was placed by a more junior doctor (2.7%) and 12 (6.6%) didn’t know the grade of the doctor carrying out the procedure. General anaesthetic was used in 84.2% (*n* = 154).

In addition to TAC placement, 28 (15.3%) women reported having progesterone supplementation at the same time, while 19 (10.4%) had an occlusion suture, where an addition suture is placed at the external cervical os, with the aim of maintaining the integrity of the cervical mucus plug and thus reducing risk of ascending infection. In women with TAC, it is usually placed after completion of the first trimester of pregnancy. Eighteen of our survey respondents with occlusion sutures had a post-TAC pregnancy, 16 of which had a successful pregnancy ending after 30 weeks’ gestation.

### Need for additional TAC

Six women had a second TAC placed due to failure of the first, either following premature ruptured membranes and/or preterm birth (*n* = 4), or because it was found, or suspected, to have pulled through or become loose (*n* = 2). In two cases it was suggested that it may have loosened due to the woman labouring with the first TAC in situ. Of the six, three had had the first TAC for trachelectomy. Four of the six women had no further pregnancies after the second TAC, while one woman had a pregnancy ending at 36 weeks, and another at 26 weeks.

### Women’s experience of seeking and receiving a TAC

Participants were invited to answer the question, in their own words: “Is there anything else you would like to tell us about your experience of have a TAC, including more details about how you came to have one, how you felt about it and any health issues or experiences you have had since?” Of all the respondents, 116 entered some text in response. The demographic characteristics of these 116 were similar to the whole cohort, as shown in Table 1. Qualitative analysis of this free text revealed the following themes: 1. the struggle to get treatment; 2. lack of knowledge amongst clinicians; 3. gratitude, hope and feeling protected and 4. possible detrimental effects of TAC.

#### Theme 1: the struggle to get treatment

A common theme noted was the struggle many had had to face in order to receive the treatment they needed. Women often had had to do their own research, and several had only found out about TAC through the Facebook group, UK TAC Support:

*“I had to investigate the cause of my loss myself and took myself privately to see my consultant who confirmed cervical incompetence.”* [ID 33]

*“Found information about the TAC on Facebook through doing research into incompetent cervix, local hospital is not very familiar with it and it does quite often need explaining.”* [ID 50]

*“I had to [do] extensive research myself, push for testing …they kindly referred me to the TAC consultant. If not for that, I would not have our son.”* [ID 155]

*“I don’t feel like my pregnancy and miscarriage were managed well… I was constantly having to try and lead my care and it made me feel very vulnerable.”* [ID 51]

When they accessed clinical care they sometimes found it inadequate and unsupportive:

*“I begged and begged to have some kind of cerclage in my next pregnancy and they reluctantly agreed [and I] had that placed at 12 weeks… [I] was given no extra appointments or care was just told to carry on as normal and they would see me at 20 weeks…[but] I didn’t even get that far before problems started.”* [ID 75]

*“Had I not found this group I would more than likely be grieving another baby as my local hospital advised a ‘wait and see’ approach following my 2 losses.”* [ID 66]

*“I saw […] privately after being told by my local obstetrician that my loss was ‘just one of these things’ and advised to ‘try again and let’s see what happens’.”* [ID 77]

*“I should have been advised of a needing a TAC from the start. I am bitterly disappointed with the care received by […] hospital, even when attending pre-term clinic they still got my treatment wrong. My file clearly outlines how much was cut with the cone biopsy and how little cervix was left.”* [ID 16]

#### Theme 2: lack of knowledge amongst clinicians

The struggle to get treatment was in part related to a lack of awareness of clinicians, of both the risks associated with invasive cervical procedures and transabdominal cerclage as a potential treatment.

*“…it does feel if you aren’t having your antenatal care at the hospital that performed your TAC then you have to fight so hard to be understood when you are at your most vulnerable.”* [ID 51]

*“[I] was told about the TAC by a mum in NICU. Had 2 consultants not willing for me to have it; I was told I needed more losses by the first, and that it was too dangerous by the second.”* [ID 49]

*“My GP knew nothing about the TAC, she looked at me blankly luckily she referred me back to my consultant from my previous pregnancies … I was lucky it was not a battle as I have heard some awful stories about GP blocking patients from being referred.”* [ID 46]

*“I really struggled with local antenatal care during my TAC pregnancy though. My local hospital team had not heard of a TAC and I was always having to explain what it was and was met with blank faces.”* [ID 51]

This lack of awareness amongst clinicians inevitably leads to inadequate information for women about the risk of preterm birth associated with cervical surgery, and how transabdominal cerclage may reduce that risk:

*“[there is] …lack of post operative information as [incompetent cervix] being a potential risk post cone biopsy. Recommendations of regular vaginal length scans on all patients who have experienced cervical surgery and then get pregnant*.” [ID 148]

*“I had had LLETZ cell removal in my 20’s … Never informed it could cause any problems in the future”* [ID 67]

*“I self advocated through my own research. I only wish the TAC was better known and woman at risk of incompetence cervix were better informed of the risks and treatment options, such as TAC before losing their babies.”* [ID 16]

*“Our original fertility consultant ignored my concerns regarding my cervix following 2 LLETZ procedures.”* [ID 122]

#### Theme 3: gratitude, hope and feeling protected

Predominantly, women who had had a successful pregnancy following the TAC felt that it had improved their experience of pregnancy and had given them hope.

*“I had given up on being a mother so knowing about the TAC gave me hope*.” [ID 145]

*“It was the only way I could have carried a baby again it gave me peace of mind to know it was the most I could do.”* [ID 5]

*“Other registrars considered the TAC to be OTT [over the top] but mentally and physically in my TAC pregnancy with the complications involved it was the best decision I made.”* [ID 11]

*“My TAC pregnancy was without any major problems and I was able to deliver a full term child which, in turn, had a profound positive impact on my physical and psychological well-being during my pregnancy and after delivery of my baby too.”* [ID 30]

*“I’m so grateful for the TAC as it’s given me 2 rainbows [live children following death of a baby].”* [ID 12]

*“The TAC gave my body the physical strength it needed and also gave me the mental strength to go through with another pregnancy.”* [ID 141]

*“It truly saved my life and the one of my last son, my TAC miracle.”* [ID 41]

*“It saved my 2 children after and I’m eternally grateful for that.”* [ID 119]

*“It was amazing to know I was protected against losing any more babies to IC [incompetent cervix] or having to have my babies fight for their lives due to premature birth.”* [ID 98]

It also gave hope to many who had not yet had a successful pregnancy:

*“I felt it was the only thing I could do to prevent any ‘what if’s’ in the future”* [ID 84]

*I now can’t get pregnant but I am so happy to have it in place for when (if) I ever manage to get pregnant again. Hopefully I can carry till later on in the pregnancy and have a living child and be a mum.* [ID 177]

*“The pregnancy with my son had been such a challenging one and I was so convinced I would not be able to carry another baby - the TAC has given me hope.”* [ID 158]

*“Even though I hadn’t had any form of cerclage, I had done my research and wanted a TAC. I felt that I wanted the best one, as I couldn’t stand the pain of losing another child.”* [ID 8]

For some, however, previous trauma was overwhelming and they remained fearful:

*“I had the TAC placed after these experiences but didn’t use it. I don’t think I could cope emotionally to try another pregnancy, even with the TAC.”* [ID 159]

*“I will not be having any more children out of fear… we are utterly traumatised.”* [ID 38]

#### Theme 4: possible detrimental effects of TAC

Although no explicit questions were asked about potential detrimental effects, some participants reported problems in free text boxes. Seven women reported ongoing pain following the TAC procedure, with speculation that this may be due to scar tissue, inflammation or endometriosis, but uncertainty remained as to whether this was directly related to the TAC. Nine women also reported changes to their menstrual periods, becoming more painful, heavier and/or irregular, and twelve expressed concern that they had been unable to conceive since having the TAC and thought this may be related.

### Pregnancies before and after TAC

**Fig. 1:**
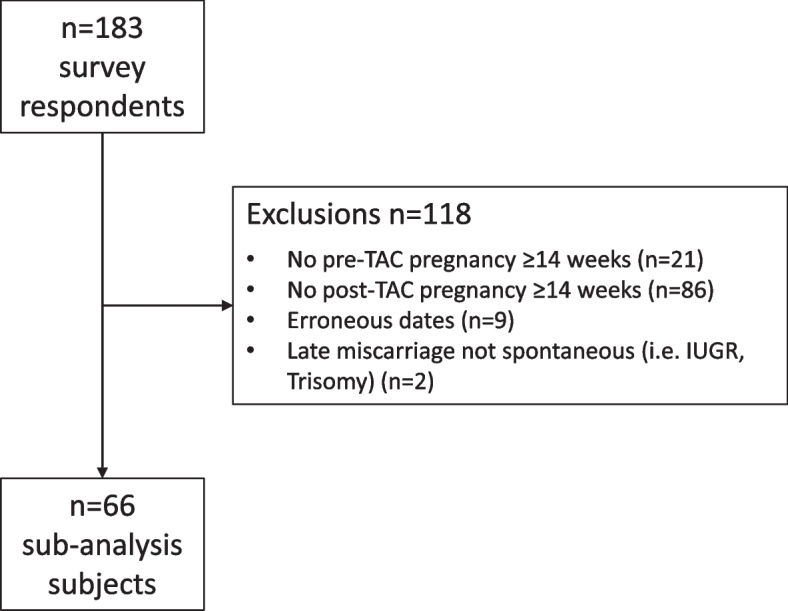
Flow chart of TAC survey respondents showing exclusions prior to sub-analysis

As a self-reported survey of women’s experience, the data collected for this study cannot be used to provide conclusive evidence of the effectiveness of this procedure. We did, however, want to compare pregnancy outcomes as reported by the participants before and after TAC placement. After exclusions, as shown in Fig. 1, 66 cases were available for sub-analysis.

Of these 66 women, 41 (62.1%) had live term births following their TAC procedure, 10 (15.2%) had their earliest baby between 34 and 36^+6^ weeks’, 6 (9.1%) between 30 and 33^+6^ weeks’, 4 (6.1%) between 24 and 29^+6^ weeks’ and 5 (7.6%) before 24 weeks’. In total, 57 women (86.4%) had a post-TAC pregnancy ending after at least 30 weeks’, a gestation at which the outcome is likely to be positive.

Unsurprisingly, our findings showed a statistically significant difference between the mean number of weeks’ gestation in pregnancies before and after TAC (-13.9, standard deviation (SD) 6.9, *p* => 0.001). Mean pre-TAC pregnancy gestation was 20.8 weeks (SD 3.61, range 14–39), while those after TAC were 34.7 (SD 5.66, range 15–39). The median number of gestation weeks gained was 15.5 (SD 6.89.

## Discussion

This is the first survey, as far as we are aware, of the experience of women with transabdominal cerclage. The vast majority of respondents (154/183, 84.1%) reported the indication for their TAC to be previous preterm birth and/or late miscarriage, with or without the additional risk factor of cervical surgery. This is unsurprising, as TAC is significantly invasive, and should not be used as a primary procedure. The need for a ‘failed cerclage’ or severe cervical trauma may be a necessary pre-requisite to justify a TAC. New research should determine ways to risk stratify, prior to pregnancy loss and in specific indications, for example, in women with a history of prior full dilatation caesarean section, where vaginally placed cerclage may be less effective [5, 6].

In addition to TAC, a number of woman also reported having the supplementary treatments progesterone (15.3%) and occlusion suture (10.4%). While there is currently little evidence to support the need for additional interventions, the difficulties of undertaking research in this area are challenging. A multicentre randomised controlled trial attempting to evaluate additional benefit of occlusion sutures concurrent with vaginally placed cerclage found no evidence of benefit, or indeed harm, but the trial had to be stopped early due to difficulties with recruitment [7]. 

Six of the 183 participants had a repeat TAC procedure, which suggests it is possible for TAC to fail, or pull out and need replacement, albeit rarely, and methods for confirming continuing stability of the cerclage should be investigated, e.g. ultrasonic evaluation. If the cerclage appears loose it may have pulled through, suggesting that the TAC should be replaced prior to a subsequent pregnancy. But it may also mean a vaginal delivery could be possible, as an alternative to caesarean section [8, 9].

This is a very high-risk group who had experienced many pregnancy losses and very preterm babies. Two reported being so traumatised by their losses that they could not face another pregnancy, even after a TAC had been placed. Whilst the majority appeared to be satisfied with the intervention, many had struggled before they received the support they needed. This is of particular concern as women from ethnic minority and socially deprived groups, who are at increased risk of poor pregnancy outcomes, may find it even harder to engage with health services [10]. The lack of knowledge about TAC amongst clinicians appeared to be not only those in General Practice, but midwives and obstetricians, which indicates a need to broaden awareness of the procedure to all those providing maternity care, as well as gynaecology services, e.g. LLETZ procedures. This could perhaps be addressed through local training, national guidelines and targeted publications.

Although our analysis comparing the outcomes of pregnancies before and after TAC cannot be taken as conclusive evidence of effectiveness, our findings concur with previous cohort studies [11, 12] and the only randomised controlled trial comparing TAC to vaginal cerclage [3].

There is currently no evidence that TAC causes long term morbidity. Shennan et al. [3] found no serious morbidities in women with TAC, although follow up was limited to the early post-operative period. There does appear to be an increased risk of cervical stenosis, which may affect fertility, in women who had a cerclage placed at the time of radical trachelectomy [13], however, Vousden et al. [14] found no difference in time to conception between women who had been randomised to TAC vs vaginal cerclage in the MAVRIC Trial (hazard ratio 1.34; 95% CI 0.72–2.50, *p* = 0.35). Nevertheless, some of our participants reported problems (i.e. pain, irregular or heavy menstrual bleeding and fertility) and whilst these cannot be directly associated with their TAC, their concerns should not go unheeded; future studies of TAC should include long term follow up and robust investigation.

As with any survey data, our findings must be considered in light of the fact that the participants were self-selected and members of support group specific for women who had struggled to find answers elsewhere. Women with satisfactory experience of clinical care are less likely to require support from such a group. Negative reported experience may be driven by a perceived need for a TAC earlier in their management. A study exploring women’s experiences utilising one-to-one interview methodology would provide richer data for more in in-depth analysis. More reliable data on variation in care and detrimental effects of TAC would be obtained through a clinical survey, such as the UK Obstetric Surveillance System (UKOSS).

## Conclusions

The experience of women with TAC is ultimately a positive one, characterised by relief and hope. But this experience follows great sadness of loss, often of more than one baby, and a difficult struggle to get the support they needed. Some of the concerns raised, particularly those of being heard and lack of awareness amongst clinicians, may be abated by NHS commissioning guidelines [15], introduced in 2019 and NHS England’s Saving Babies Lives Care Bundle [16] with their recommendations for referring women at risk to specialist preterm services.

### Supplementary Information

**Additional file 1.**

## Data Availability

The datasets generated during and/or analysed during the current study are available from the corresponding author on reasonable request.

## Abbreviations

LLETZ: Large loop excision of the transformation zone
LM: Late miscarriage
NHS: National Health Service
PPI: Patient and public involvement
PTB: Preterm birth
TAC: Transabdominal cerclage

## References

[1] Blencowe H, Cousens S, Oestergaard MZ, Chou D, Moller AB, Narwal R, Adler A, Garcia CV, Rohde S, Say L, Lawn JE (2012). National, regional, and worldwide estimates of preterm birth rates in the year 2010 with time trends since 1990 for selected countries: a systematic analysis and implications. Lancet.

[2] Alfirevic Z, Stampalija T, Medley N (2017). Cervical stitch (cerclage) for preventing preterm birth in singleton pregnancy. Cochrane Database Syst Rev.

[3] Shennan A, Chandiramani M, Bennett P, David AL, Girling J, Ridout A, Seed PT, Simpson N, Thornton S, Tydeman G, Quenby S, Carter J. MAVRIC: a multicenter randomized controlled trial of transabdominal vs transvaginal cervical cerclage. Am J Obstet Gynecol. 2020;222(3):261–e1. 10.1016/j.ajog.2019.09.040 31585096

[4] Milne J, Oberle K (2005). Enhancing rigor in qualitative description. J Wound Ostomy Continence Nurs.

[5] Watson HA, Carter J, David AL, Seed PT, Shennan AH (2017). Full dilation cesarean section: a risk factor for recurrent second-trimester loss and preterm birth. Acta Obstet Gynecol Scand.

[6] Hickland MM, Story L, Glazewska-Hallin A, Suff N, Cauldwell M, Watson HA, Carter J, Duhig KE, Shennan AH (2020). Efficacy of transvaginal cervical cerclage in women at risk of preterm birth following previous emergency cesarean section. Acta Obstet Gynecol Scand.

[7] Brix N, Secher NJ, McCormack CD, Helmig RB, Hein M, Weber T, Mittal S, Kurdi W, Palacio M, Henriksen TB, CERVO group (2013). Randomised trial of cervical cerclage, with and without occlusion, for the prevention of preterm birth in women suspected for cervical insufficiency. BJOG.

[8] Carlisle NH, Ridout AE, Shennan AH. Successful vaginal delivery following an abdominal cerclage removal in pre-term labour. Int J Obstet Gynaecol Stud. 2017;1(1):1–2.

[9] Vandermolen BI, Hezelgrave NL, Carter J, Shennan AH (2016). Successful vaginal delivery following an abdominal cerclage, predicted by serial vaginal cervical ultrasound. J Obstet Gynaecol.

[10] Kapadia D, Zhang J, Salway S, Nazroo J, Booth A, Villarroel-Williams N, Becares L, Esmail A. Ethnic inequalities in healthcare: a rapid evidence review. NHS Race and Health Observatory. 2022. Online. Available at: https://www.nhsrho.org/publications/ethnic-inequalities-in-healthcare-a-rapid-evidence-review/.

[11] Davis G, Berghella V, Talucci M, Wapner RJ (2000). Patients with a prior failed transvaginal cerclage: a comparison of obstetric outcomes with either transabdominal or transvaginal cerclage. Am J Obstet Gynecol.

[12] Fick AL, Caughey AB, Parer JT (2007). Transabdominal cerclage: can we predict who fails?. J Matern Fetal Neonatal Med.

[13] Li X, Li J, Wu X (2015). Incidence, risk factors and treatment of cervical stenosis after radical trachelectomy: a systematic review. Eur J Cancer.

[14] Vousden NJ, Carter J, Seed PT, Shennan AH (2017). What is the impact of preconception abdominal cerclage on fertility: evidence from a randomized controlled trial. Acta Obstet Gynecol Scand.

[15] UK Preterm Clinical Network. Reducing preterm birth: guidelines for commissioners and providers. 2019. Available at: https://www.tommys.org/sites/default/files/Preterm%20birth%20guidelines.pdf.

[16] NHS England. Saving babies’ lives version 2: a care bundle for reducing perinatal mortality. NHS England; 2019. Available at: https://www.england.nhs.uk/wp-content/uploads/2019/07/saving-babies-lives-care-bundle-version-two-v5.pdf.

