# Supplementary material for: “I had given up on being a mother”: a survey of 183 women’s experience of transabdominal cerclage (TAC)
Source: BMC Pregnancy Childbirth. 2023 Oct 24;23:751. doi: 10.1186/s12884-023-06001-w (PMC10599032; doi:10.1186/s12884-023-06001-w)
Supplement: Supplementary file 1 — Additional file 1. [file 12884_2023_6001_MOESM1_ESM.pdf]

Online survey for UK TAC Support – 14 11 2019

You are being invited to participate in a research study titled "Women's experience of transabdominal cerclage (TAC)"***.*** This study is being carried out by Professor Andrew Shennan and Jenny Carter from King's College London and Joanne Deery who is facilitator of the UK TAC Support group. We want to learn, from women who have a TAC, about their experience of transabdominal cerclage and their pregnancies before and afterwards. The survey may take you approximately 20-30 minutes to complete. Your participation in this study is entirely voluntary. Please click on the link below for the participant information sheet. If you are happy to proceed, please confirm this by ticking the consent box.

1. About your transabdominal cerclage (TAC):

    a. When was your transabdominal cerclage carried out? (MM/YY)
    b. At which hospital was it carried out? (free text)
    c. Was the doctor who carried it out a Consultant? (yes/no/don't know)
    d. Why did you have a transabdominal cerclage? (tick boxes: Previous late miscarriage/s or preterm birth/s; Cervical surgery e.g. following an abnormal smear test; Other). If "cervical surgery…" type (Cone, LLETZ, Laser, Trachelotomy, Unsure, MM/YY), if "other" – free text box.
    e. Did you have the operation before or while you were pregnant? (before pregnancy, during pregnancy)
    f. What type of operation – open procedure *(explanatory text)*/laparoscopic *(explanatory text)* (exclusive tick options).
    g. Were you asleep or awake for the operation? (exclusive tick options)

2. Have you had more than one transabdominal cerclage (TAC) operation? (Yes/No)

    If Yes, (repeat questions as above), if No go to Q.3

3. Please tell us about all your pregnancies before and after your TAC (including early and late miscarriages and terminations) *(one row for each, added as needed)*:

    a. MM/YY
    b. Hospital (free text)
    c. Outcome of pregnancy (miscarriage/termination/stillbirth/live birth)
    d. How many weeks of pregnancy? 0-44
    e. For miscarriages: (all happened naturally/needed medication to complete the process/needed an operation)
    f. For terminations: (medication/operation)
    g. Onset of labour: (Spontaneous/Induction/No labour)
    h. Mode of delivery: (Normal birth/Ventouse/Forceps/Caesarean).
    i. For pregnancy losses and/or preterm deliveries, did the doctors looking after you give you a reason why this happened? (free text box)
    j. Any complications or comments (e.g. infection, long hospital stay): (free text box)

4. Have you ever had a caesarean section in labour? (Yes/No)

If Yes, please indicate *(one row for each, added as needed)*, if No, go to Q.5

a. Which pregnancy: MM/YY
b. Did you have the caesarean when you were…(in early labour (up to 3 cm), first stage (4-9 cm), second stage (10 cm or fully dilated), Not sure).
c. If you can remember, please enter exact number of centimetres you were when you had your CS: (0-10)

5. Did you have a vaginal cerclage in any of your pregnancies? (Yes/No)

   If yes, please indicate *(one row for each, added as needed)*, if No, go to Q.6

   a. Which pregnancy: (MM/YY)
   b. What type of cerclage was it?; (high/Shirodkar; low/McDonalds; Don't know)
   c. Was is placed before a change in your cervical length or afterwards (*because your cervix was getting shorter*)? (Before change in cervical length/After change in cervical length/Don't know)
   d. Was it an emergency (sometimes called "Rescue") cerclage, which is placed when the membranes are visible vaginally? (Yes/No/Don't know)
   e. When was the cerclage placed? (No. of weeks of pregnancy/Not sure).
   f. When was it removed? (No. of weeks of pregnancy/Not sure).

6. Did you take progesterone in any of your pregnancies? (Yes/No).

   If yes, please indicate *(one row for each, added as needed),* if No, go to Q.7

   a. Which pregnancy: (MM/YY)
   b. How did you take it?; (Vaginally/ Rectally/ By injection)
   c. At how many weeks of pregnancy did you start taking it? (No. of weeks of pregnancy/Can't remember)
   d. At how many weeks of pregnancy did you stop taking it? (No. of weeks of pregnancy/Can't remember)

7. Did you have any other treatments to reduce your risk of preterm birth in any of your pregnancies (e.g. occlusion suture, bedrest, Arabin pessary)? (Yes/No)

   If yes, please indicate *(one row for each, added as needed)*, if No, go to G.8

   a. Which pregnancy? MM/YY
   b. What treatment was it? (*drop down list and free text box for "Other"*)
   c. At how many weeks of pregnancy did the treatment start: (No. of weeks of pregnancy/Can't remember)
   d. At how many weeks of pregnancy did the treatment stop: (No. of weeks of pregnancy/Can't remember)

8. About you:

a. How old are you? (Age)
b. Which of the following most closely matches your ethnicity: (Asian/Black/Mixed/White/Other).
c. Height (ft or cm)
d. Weight (stones/lb or kg).

9. Is there anything else you would like to tell us about your experience of having a TAC, including more details about how you came to have one, how you felt about it and any health care issues or experiences you have had since? (big free text box)

Thank you for taking part in this survey. Your answers will help us to understand more about women's experience of transabdominal cerclage.
